# Accelerated calciprotein crystallization time (T50) is correlated with impaired lung diffusion capacity in systemic sclerosis

**DOI:** 10.3389/fimmu.2024.1425885

**Published:** 2024-09-27

**Authors:** Marija Geroldinger-Simic, Azmat Sohail, Mehdi Razazian, Beatrice Krennmayr, Victoria Pernsteiner, Thomas Putz, Helmut K. Lackner, Andreas Pasch, Norbert Sepp, Ioana Alesutan, Jakob Voelkl

**Affiliations:** ^1^ Department of Dermatology and Venereology, Ordensklinikum Linz Elisabethinen, Linz, Austria; ^2^ Faculty of Medicine, Johannes Kepler University, Linz, Austria; ^3^ Institute for Physiology and Pathophysiology, Johannes Kepler University Linz, Linz, Austria; ^4^ Division of Physiology and Pathophysiology, Otto Loewi Research Center for Vascular Biology, Immunology and Inflammation, Medical University of Graz, Graz, Austria; ^5^ Calciscon AG, Biel, Switzerland; ^6^ Department of Nephrology and Medical Intensive Care, Charité-Universitätsmedizin Berlin, Corporate Member of Freie Universität Berlin and Humboldt Universität zu Berlin, Berlin, Germany; ^7^ DZHK (German Centre for Cardiovascular Research), Partner Site Berlin, Berlin, Germany

**Keywords:** mineral buffering, serum calcification propensity, calciprotein particles, systemic sclerosis, phosphate

## Abstract

**Background:**

Systemic sclerosis (SSc) is a complex auto-immune disease characterized by vascular damage, inflammation, fibrosis and calcinosis, where pulmonary involvement is the leading cause of mortality. Calciprotein particles (CPPs) are increasingly formed upon disbalance of the physiological mineral buffering system and induce pro-inflammatory effects. This exploratory study investigated whether functional indicators of the endogenous mineral buffering system are dysregulated in SSc and linked to disease activity.

**Methods:**

T50 (calciprotein crystallization test or serum calcification propensity) and hydrodynamic radius of secondary CPPs (CPP2) were determined in serum samples from 78 SSc patients and 44 controls without SSc, and were associated with disease activity markers of SSc.

**Results:**

T50 was reduced and CPP2 radius was increased in SSc patients as compared to controls, indicating a deranged mineral buffering system. This was accompanied by slightly higher serum phosphate and PTH levels in SSc patients, while iFGF23 was not significantly modified. Longitudinally, all parameters remained unchanged over time in SSc patients, only iFGF23 increased. While the modified Rodnan skin score showed some inconsistent correlations with mineral buffering indicators, their association was not independent of other factors. However, lower T50 was significantly correlated to reduced lung diffusion capacity and this association remained significant in a multivariate linear regression model.

**Conclusion:**

This study provides indications for a disturbed mineral buffering system in SSc. Increased serum calcification propensity (lower T50) is correlated with impaired lung diffusion capacity, suggesting a possible role of deranged mineral buffering in disease progression. Further studies are required to confirm these observations in larger cohorts and to investigate a putative functional relevance.

## Introduction

Systemic sclerosis (SSc) is a complex autoimmune rheumatic disease characterized by multi-organ involvement, due to vascular damage, inflammation, fibrosis and calcinosis (1, 2). Mortality in SSc is mainly caused by pulmonary and cardiac involvement (3). The mechanisms underlying the onset and progression of SSc are incompletely understood.

In the general population, disordered phosphate homeostasis has been linked to increased mortality due to cardiovascular and pulmonary causes (4). But the association of hyperphosphatemia and mortality has been most extensively studied in chronic kidney disease (CKD) (5). In CKD, reduced glomerular filtration of phosphate is initially counteracted by the phosphaturic hormones fibroblast growth factor 23 (FGF23) and parathyroid hormone (PTH), before these compensatory mechanisms are overwhelmed and hyperphosphatemia ensues (5). The insights from these patients also sparked research into a physiological mineral buffering system. Calcium and phosphate levels in serum are considered close to supersaturation (6), but ectopic formation of calcium-phosphate crystals is prevented by local and systemic crystallization inhibitors (7). A decisive role in this is attributed to the protein Fetuin-A, which scavenges calcium-phosphate ion clusters to form calciprotein monomers (8). These are able to aggregate and form primary calciprotein particles (CPP1), which can further mature into secondary CPP2 (8). Especially in the cardiovascular system (9), CPP2 are discussed to mediate the toxic effects of phosphate on cells, which has already been termed mineral stress (10).

Accordingly, indicators of serum mineral buffering capability are closely linked to mortality (10). A rather novel assessment is the hydrodynamic radius of CPP2, which may be indicative of the strength of the mineral buffering system and a putative marker for mineral stress (11). CPP2 radius is linked to mortality in peripheral artery disease (12) and dialysis patients (11). But the most established test indicative of serum mineral buffering capability is the serum calciprotein crystallization test (T50, also known as serum calcification propensity test), determined by the transformation time from CPP1 to CPP2 upon adding excessive calcium/phosphate to serum (13). The association of low T50 (faster transformation time) with increased mortality in CKD has been well established, and recently extended to the general population (14, 15). The mechanisms underlying this association are currently unclear, but might involve the propagation of a more inflammatory state during calcium-phosphate stress (7). Accordingly, low T50 is linked to inflammatory markers (16).

Due to the close connection of T50 and mortality, putative therapeutic approaches targeting T50 are discussed (17, 18) and measurements were extended to populations beyond CKD, such as diabetes (19), aldosteronism (20) or heart failure (21). Moreover, T50 has also been associated with disease activity in systemic lupus erythematosus (22) and pseudoxanthoma elasticum (23). Some previous observations indicated a possible dysregulation of mineral homeostasis in SSc (24, 25). Thus, we conducted an exploratory study to investigate alterations of the mineral buffering system and its possible relevance in SSc.

## Materials and methods

### Study cohort and clinical data

This exploratory study group included 78 patients with SSc and 44 controls without SSc, which were recruited at the Department of Dermatology, Ordensklinikum Linz, Austria. All study participants provided written informed consent. The study has been approved by the Ethics Committee of the Johannes Kepler University Linz, Austria (protocol 1265/2019 and amendments). Patients were assessed and included in the study during routine annual checkups (laboratory, serological tests, and assessment of organ involvement). Diagnosis of SSc was performed following the 2013 classification criteria for SSc by the American College of Rheumatology (ACR) and the European League Against Rheumatism (EULAR). The inclusion criteria for patients were diagnosis of SSc and age 18–90. Exclusion criteria for control group were known acute infections, liver and/or kidney diseases and diabetes mellitus. Blood sampling was performed during routine clinical controls. Where available, routine laboratory parameters and clinical history were documented and eGFR was calculated by the CKD-EPI 2021 equation (26). The modified Rodnan skin score (mRSS) determined by a single investigator was used for the measurement of skin fibrosis. Lung diffusion measurements (diffusing capacity of lung carbon monoxide, DLCO) were not conducted during each time-frame of sample draw, thus one timepoint was investigated within one year of study inclusion. When two measurements of DCLO were available, the timepoint closest to the blood collection was selected.

### Serum calcification propensity and CPP2 size

Serum calcification propensity (7) measured as half-maximal time of transformation (T50) from CPP1 to CPP2 was performed at the reference laboratory of Calciscon (Switzerland) (27). Also, dynamic light scattering was utilized to measure CPP2 hydrodynamic radius as previously described (21, 28). Serum samples were frozen at -80°C until measurements without freeze-thaw cycles.

### ELISAs and zinc measurements

Serum samples from the study cohort were analyzed by ELISA for intact FGF23 (Quidel) and 1-84 PTH (Quidel) according to the manufacturer’s instructions. Zinc was determined by inductively coupled plasma mass spectrometry.

### Statistical analysis

Data from patients are shown as Median and 25-75^th^ percentile unless otherwise indicated. Group comparisons were performed by paired or unpaired student t-test, Mann-Whitney test or Wilcoxon matched pair signed rank test, as indicated by Shapiro-Wilk test for normality distribution. Spearman correlation test was performed for correlations. For further investigations, a linear regression model was used where mRSS or DLCO cSB was defined as the dependent variable. In the regression model age, sex, calcium, phosphate, eGFR, disease duration, height, weight, T50 and CPP2 radius were included as variables. P values of <0.05 were considered statistically significant.

## Results

To investigate alterations in the mineral buffering system in SSc, we recruited SSc patients and suitable controls. Median disease duration as years since first diagnosis of SSc was 6 (2–12) years. Further information on SSc patients and controls is shown in **Supplementary Tables S1**, **S2**. As shown in **Table 1**, SSc patients and controls were of similar distributions with minor differences in age and sex, and no significant difference in circulating concentrations of calcium, magnesium or eGFR were observed. Zinc serum levels were decreased in SSc patients (**Table 1**). Compared to the controls, we observed an elevated plasma phosphate concentration in the SSc patients (**Table 1**), which was still within the standard reference range (29). In addition, PTH concentrations were significantly increased, but an increase of iFGF23 did not reach statistical significance (p=0.0865).

**Table 1:** Characteristics of the study cohort.

	CTR	SSc	N
Sex [n female subj.]	29	63	44/78
Age [yrs]	57 (48-63)	61 (53-67)	44/78
Calcium [mmol/l]	2.34 (2.30-2.41)	2.36 (2.32-2.42)	33/70
Phosphate [mmo/l]	0.93 (0.86-1.06)	1.10 (1.00-1.20)***	32/71
Magnesium [mmol/l]	0.86 (0.82-089)	0.87 (0.81-0.90)	24/34
Zinc [ug/l]	857.6 (747.2-926.7)	742.2 (661.9-848.2)**	43/77
eGFR [ml/min/1.73m^2^]	97.7 (89.0-104.8)	95.3 (80.1-102.1)	42/77
intact FGF23 [pg/ml]	5.84 (3.65-10.55)	7.65 (3.79-18.08)	44/76
1-84 PTH [pg/ml]	12.07 (7.77-21.63)	17.12 (10.46-27.01)*	44/77

Sex (shown as n of female subjects) as well as median and interquartile range of age, eGFR, serum levels of calcium, phosphate, magnesium, intact FGF23 and 1-84 PTH in controls and SSc patients. *,**, *** indicates p< 0.05, p<0.01, p<0.001 vs. CTR.

**Figure 1:**
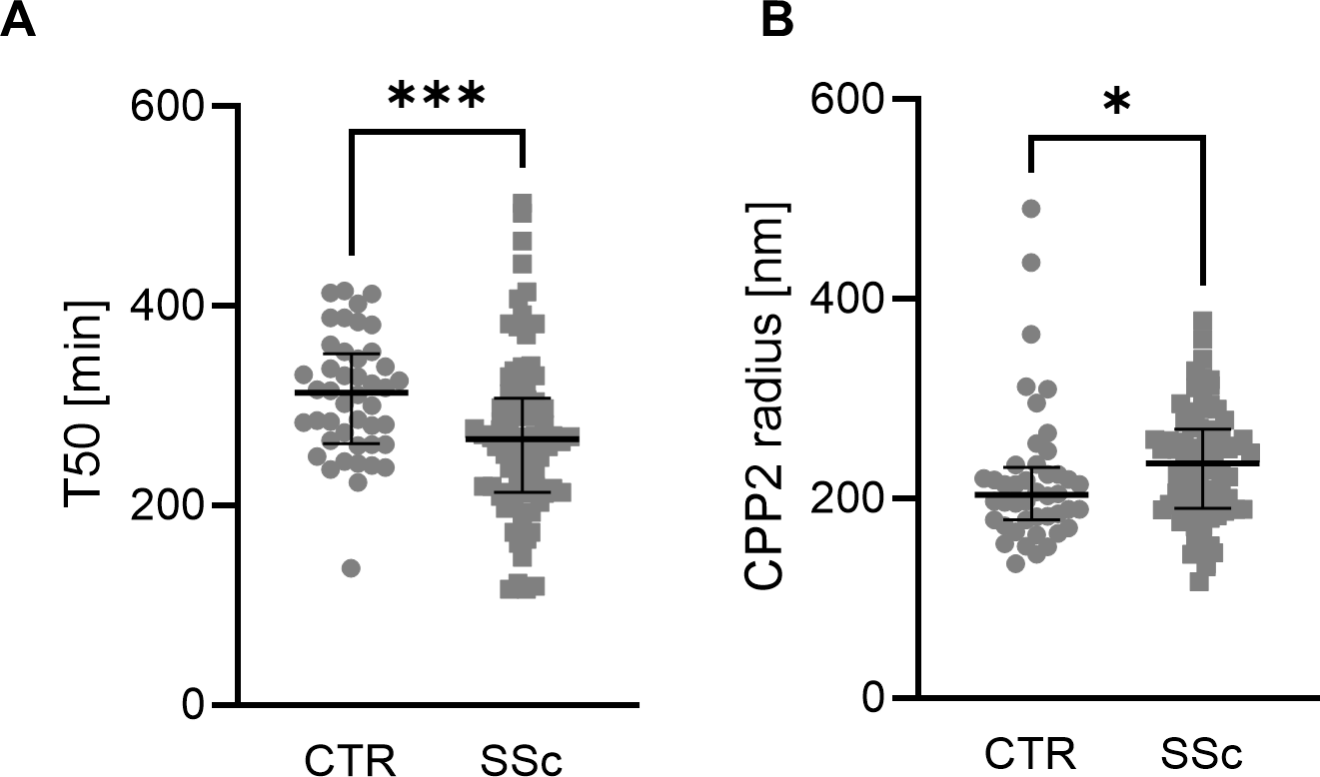
Mineral stress markers in SSc patients. Individual measurements with median and interquartile range (n=44-78) of T50 **(A**, Mann-Whitney test) and CPP2 size (hydrodynamic radius, **B**, Mann-Whitney test) in controls (CTR) and patients with SSc. *, *** indicates p< 0.05, p<0.001 respectively.

Most importantly, we observed a significantly lower T50 in SSc patients (**Figure 1A**), indicating higher serum calcification propensity. In addition, hydrodynamic radius of CCP2 was increased in SSc patients (**Figure 1B**).

**Figure 2:**
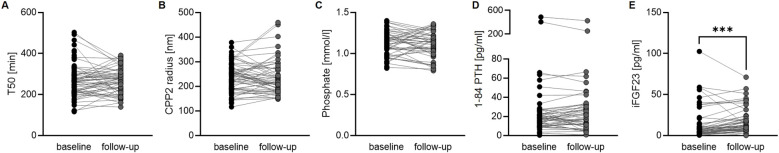
Alterations of mineral metabolism over time. Measurements of T50 (**A**, n= 54, Wilcoxon matched pair signed rank test) and CPP2 size (hydrodynamic radius, **B**, n= 54, Wilcoxon matched pair signed rank test) as well as phosphate (**C**, n= 46, paired t-test), 1-84 PTH (**D**, n=50, Wilcoxon matched pair signed rank test) and iFGF23 (**E**, n=51, Wilcoxon matched pair signed rank test) in patients with SSc at baseline and at follow-up. *** indicates p<0.001.

We then investigated the alterations of mineral homeostasis in serum of SSc patients over time. The median time difference between the 2 measurements was 220 days (IQR: 180-286). Between baseline and follow-up, T50 and CPP2 radius mostly remained stable and no significant differences were observed (**Figures 2A, B**). Also, PTH or phosphate concentrations showed no significant alterations over time (**Figures 2C, D**). However, serum levels of FGF23 significantly increased from baseline to follow-up (**Figure 2E**).

**Figure 3:**
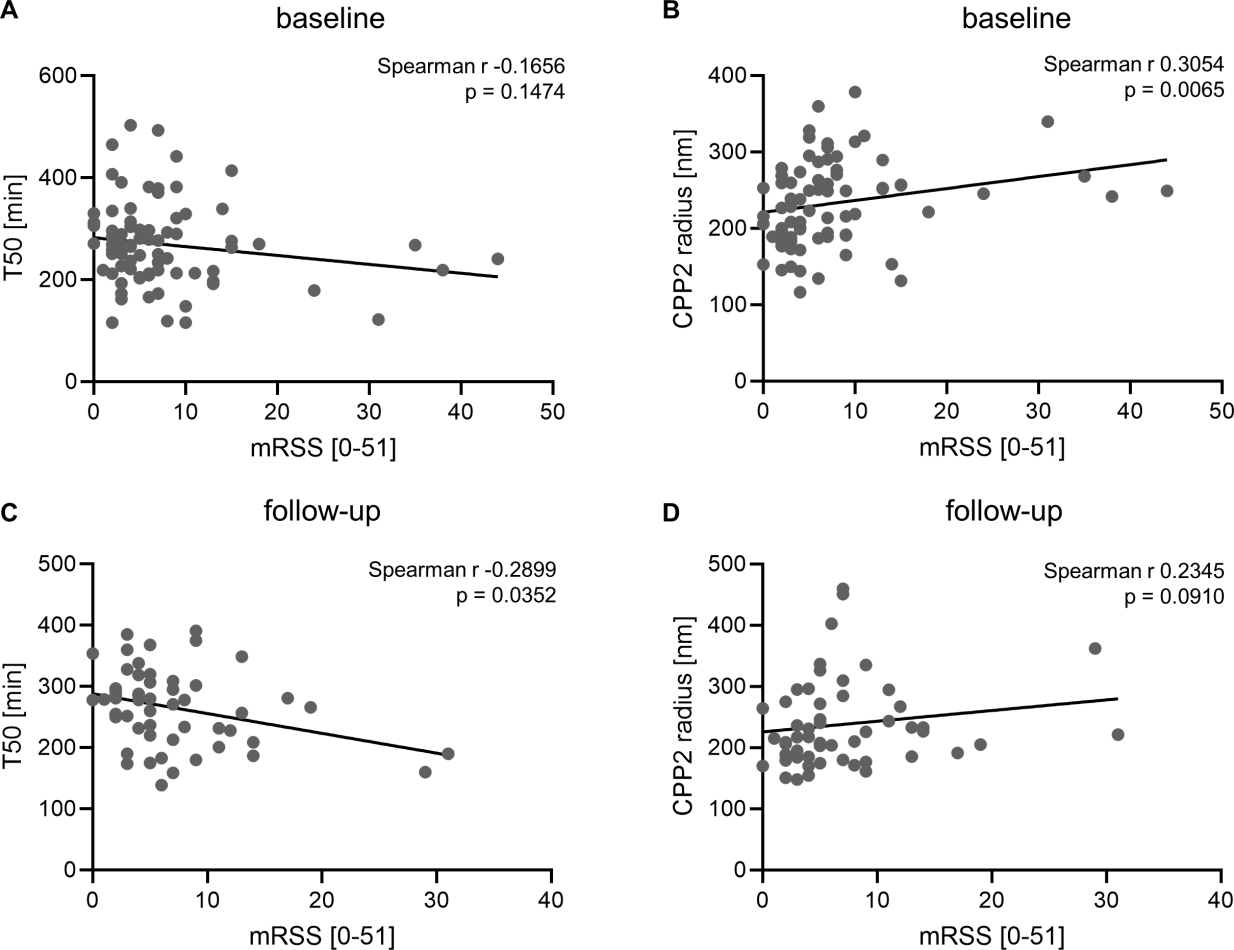
Association of mineral stress markers with modified Rodnan skin score. Correlation of serum calcification propensity T50 (**A**, n=78) or CPP2 size (hydrodynamic radius, **B**, n=78) with modified Rodnan skin score in SSc patients at baseline. Correlation of serum calcification propensity T50 (**C**, n=53) or CPP2 size (hydrodynamic radius, **D**, n=53) with modified Rodnan skin score in SSc patients at follow-up. P value is indicated in the figure.

Next, we investigated an interaction of mineral buffering indicators with the skin thickness measured by mRSS in the SSc patients (**Figures 3A, B**) in the baseline samples (mRSS Median 5.5, IQR 3.0-9.0). No significant correlation of mRSS with T50 (Spearman r -0.1656, p=0.1474, n=78) was observed. However, a significant correlation of CPP2 radius and mRSS emerged (Spearman r 0.3054, p=0.0065, n=78). A linear regression model with mRSS as dependent variable and including age, sex, calcium, phosphate, eGFR, disease duration, height, weight, T50 and CPP2 radius showed no significant effects for either T50 or CPP2 radius (**Supplementary Table S3**). To further investigate mineral homeostasis dysregulation and mRSS, we repeated the analysis in the measurements obtained from follow-up samples (**Figures 3C, D**). In these samples, a significant correlation of mRSS with T50 was observed (Spearman r -0.2899, p=0.0352, n=53). In the follow up samples, the correlation of CPP2 radius with mRSS did not reach statistical significance (Spearman r 0.2345, p = 0.0910, n=53). However, in the linear regression model neither T50 nor CPP2 radius showed significant effects (**Supplementary Table S4**).

In addition, we stratified patients into group with and without calcinosis cutis (CC). Between patients with and without known CC, we found no difference in T50 (CC: Median 266.5 min (213.0-331.5), n=30; no CC: Median 268.0 min (212.0-306.0), n=47; p=0.4416) or CPP2 hydrodynamic radius (CC: Median 225.8 nm (186.6-257.4), n=30; no CC: Median 238.8 nm (190.7-278.7), n=47; p=0.4149).

**Figure 4:**
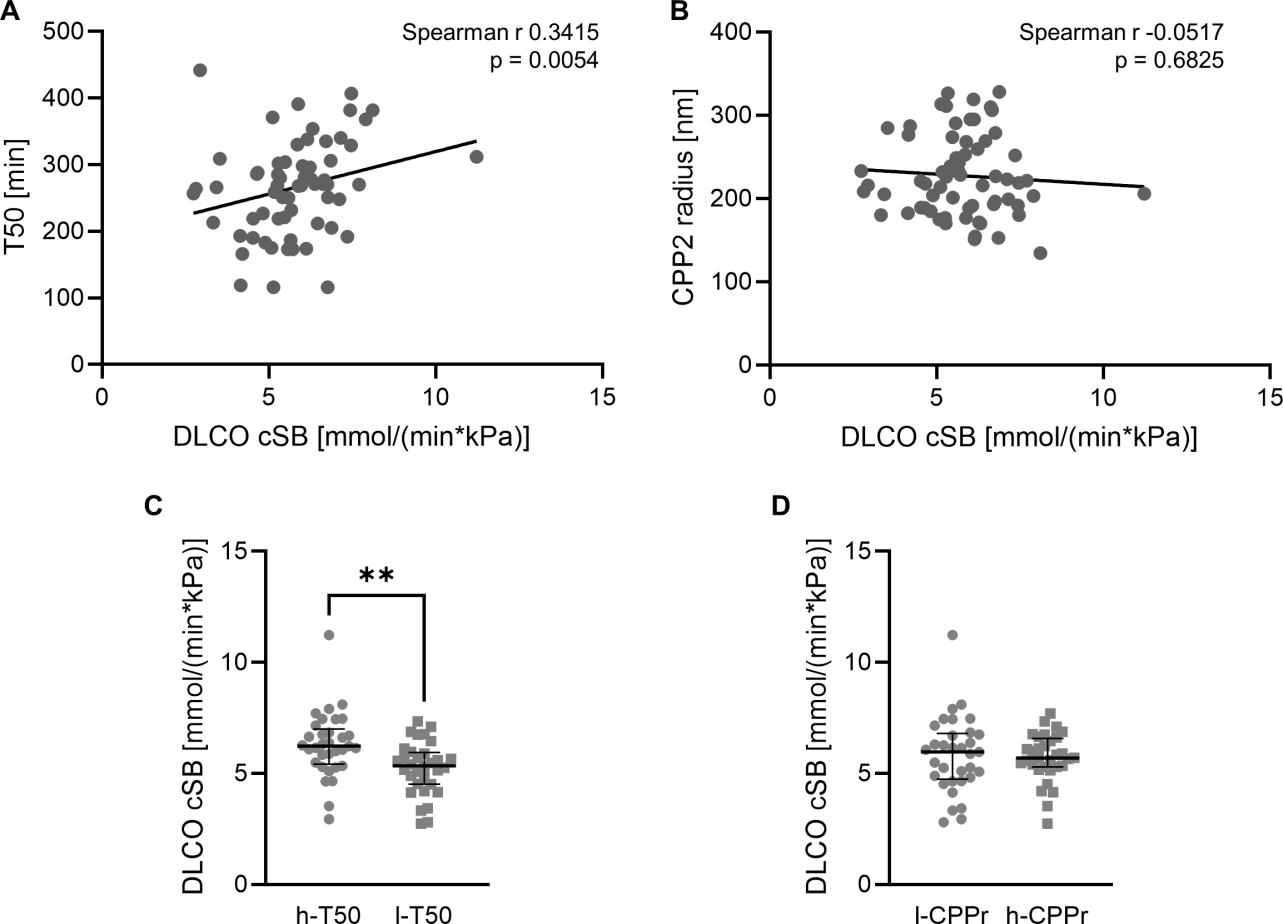
Association of mineral stress markers with pulmonary function. Correlation of serum calcification propensity T50 (**A**, n=65) or CPP2 size (hydrodynamic radius, **B**, n=65) with lung diffusion capacity DLCO cSB (mmol/(min*kPa) in SSc patients. P value is indicated in the figure. Median and interquartile range of DLCO cSB (mmol/(min*kPa) in SSc patients stratified into high (h-T50, n=33, equal to or above the median of T50) and low (l-T50, n=32, below the median of T50) T50 (**C**, Mann-Whitney test) or stratified into low (l-CPPr, n=33, equal to or below the median of CPP2 radius) and high (h-CPPr, n=32, above the median of CPP2 radius) CPP2 hydrodynamic radius (**D**, unpaired t-test). ** indicates p<0.01.

We then explored the role of dysregulated mineral buffering as factor in pulmonary function of SSc patients. To this end, we investigated the correlation of T50 and lung diffusion results collected from routine clinical assessment of SSc patients. As shown in **Figure 4A**, we observed a significant correlation of T50 and the diffusing capacity of the lungs for carbon monoxide (DLCO cSB, Spearman r 0.3415, p=0.0054, n=65). No correlation was observed for DLCO and CPP2 radius (Spearman r -0.0517, p=0.6825, n=65, **Figure 4B**). We then again utilized a linear regression model with DLCO as dependent variable and included T50, CPP2 radius, age, sex, calcium, phosphate, eGFR, disease duration, height and weight as variables. This revealed only T50 and age as significantly associated with DLCO (**Table 2**) in this cohort. Patients were then stratified into 2 groups according to the median of T50 or CPP2 radius. DLCO was significantly reduced in the low T50 group vs high T50 group, but no difference was observed between the two groups stratified by CPP2 radius (**Figures 4C, D**).

**Table 2:** Association of T50 with lung diffusion capacity.

DLCO cSB [mmol/(min*kPa)]	beta	p
Calcium [mmol]	0.152	0.216
Phosphate [mmol]	-0.059	0.617
eGFR [ml/min/1.73m^2^]	0.090	0.478
Disease duration [yrs]	0.094	0.429
Body weight [kg]	0.164	0.173
Body size [cm]	0.178	0.309
Sex [m0/f1]	0.036	0.838
**Age [yrs]**	**-0.479**	**0.001**
**T50 [min]**	**0.361**	**0.010**
CPP2 radius [nm]	0.180	0.204

Factors associated with lung diffusion capacity, DLCO cSB (mmol/(min*kPa), n=57, change in F p=0.001) in patients with systemic sclerosis in a linear regression model, shown with regression coefficients (beta) and levels of significance (p value; bolded if p < 0.05).

Additional analyses were then conducted to indicate determinants of T50 in SSC patients. In the control probands, T50 was not significantly correlated to calcium (Spearman r 0.1307, p=0.4686, n=33), zinc (r 0.1166, p=0.4565, n=43), age (r -0.1710, p=0.2671, n=44), eGFR (r 0.06248, p=0.6943, n=42), FGF23 (r 0.008461, p=0.9565 n=44) or PTH (r 0.2553, p=0.0944, n=44), but showed a correlation with phosphate (r -0.4147, p=0.0183, n=32). In SSc patients, T50 was not significantly correlated to calcium (r 0.09540, p=0.4321, n=70), age (r -0.02944, p=0.7981, n=78), eGFR (r -0.05273, p=0.6488, n=77), FGF23 (r 0.01691, p=0.8848 n=76) or PTH (r 0.1078, p=0.3506, n=77), but showed a correlation with phosphate (r -0.2931, p=0.0131, n=71) and zinc (r 0.3282, p=0.0036, n=77). Similar observations were made at the follow-up measurements, where T50 again correlated with phosphate (r -0.4113, p=0.0027, n=51), zinc (r 0.3640, p=0.0068, n=54) and also with FGF23 (r -0.2877, p=0.0406, n=51).

## Discussion

The current study provides indication for a dysregulation of mineral buffering in SSc and further suggests its putative pathophysiological relevance. In our cohort, SSc patients exhibited lower T50, conjoined with an increased size of CPP2. T50 reflects the sum of promoters and inhibitors of calcium-phosphate crystal formation in an individual serum sample (30). An increased formation of CPPs may induce pro-inflammatory effects and pathological mechanisms in cells (also termed mineral stress (10)), as was discussed for rheumatoid arthritis (31) or hypoxic fibrosis (32). Another indicator of the mineral buffering system strength is the hydrodynamic radius of CPP2, which may be distinct from T50 in its mechanisms and interpretation (11).

In theory, a deranged mineral buffering system in SSc may be of relevance for the course of the disease. This concept was investigated by the association of mineral buffering indicators with disease activity markers. While we observed some correlations of mineral buffering indicators with mRSS, these associations remained rather inconclusive in multivariate models. Also, we did not find any clear indications that mineral buffering indicators might be altered depending on the presence of known calcinosis cutis. But a clear effect was observed for T50 and DLCO cSB, indicating that a low T50 is linked to a reduced lung diffusion capacity. DLCO is an important surrogate marker for interstitial lung disease progression in SSc (33). Moreover, reduction of DLCO has been suggested as an early sign of lung involvement in SSc (34). In turn, CPPs have been associated with pro-fibrotic effects in the fetuin-A-deficient mouse (35) and murine renal fibrosis (32). In the human kidney, T50 was independently associated with interstitial fibrosis according to histological changes (36). Indications for renal fibrosis were observed in mice after excessive phosphate feeding (37). In rats with a subtotal nephrectomy, phosphate binding reduced CPPs in serum along with renal inflammation and fibrosis (38). Although much less studied beyond kidney disease, CPPs might mediate pathological effects in other conditions (32). Formation of calcium-phosphate crystals in the alveolar lumen is also able to cause pulmonary fibrosis and hypertension (39). Most importantly, phosphate directly increases inflammation in bronchial epithelium (40). Hyperphosphatemic klotho-deficient mice develop airway inflammation and a phenotype of COPD (41). Fetuin-A, a key component of CPPs, has been identified as a predictor for exacerbations in COPD (42). Furthermore, phosphate has been discussed as a putative factor in CKD-associated pulmonary diseases (43). Thus, there are some indications of a putative link between mineral buffering homeostasis and mineral stress as indicated by T50 with inflammatory and fibrotic processes in the lung. Although a direct inflammatory effect of CPPs on the lung in SSc might be plausible, the current data is observational and no causality can be interpreted. Clearly, further research is required to study the link between pulmonary alterations, CPPs and T50 as well as their role in SSc.

An important determinant of increased serum calcification propensity (lower T50) are higher phosphate concentrations (44). Rather surprisingly and without any differences in eGFR or calcium, phosphate levels in SSc patients were slightly, but significantly elevated as compared to controls. Although comparatively higher, phosphate levels remained in the normal range and stable during the observation time. Similar to our observations, increased phosphate levels were also described in an Iranian SSc cohort (24) as well as a Spanish cohort (45). Thus, the slightly increased phosphate levels in SSc do not appear an isolated finding in our cohort, but may be a previously unrecognized pathophysiological aspect of SSc. While we did not measure vitamin D levels in our cohort, vitamin D levels are typically reduced in SSc (46) and therefore might not be the primary target to explain the elevated phosphate levels. An increase of PTH was observed in our SSc patients, as also indicated by some previous observations (47, 48). FGF23 was not significantly increased in SSc versus controls, but increased over time in serum of SSc patients. With the rather short follow-up in a small and not completely homogenous cohort of SSc, the general alterations of FGF23 and putative underlying mechanisms cannot be identified in this cohort. Descriptions of FGF23 in SSc patients have been inconsistent, as reduced FGF23 levels (25), increased FGF23 (49) or no difference (24, 50) compared to healthy controls were described. However, reductions of circulating klotho were previously also described (24, 51) and could be indicative of a reduced phosphaturic effect of FGF23 in the kidney. In addition, zinc levels appeared to be reduced in our SSc patients. Some previous studies hinted at reduced zinc levels in SSc (52). Zinc has been shown to inhibit calcific processes (11, 53, 54) and higher serum zinc was linked to improved serum calcification propensity (30, 55, 56). Therefore, high phosphate and low zinc concentrations could favor an increased serum calcification propensity in SSc. Overall, further research is required to identify the SSc-specific alterations of mineral homeostasis in larger and multi-centric studies as well as the underlying causes for elevated phosphate levels and reduced T50 in SSc.

Our study is limited by its exploratory setting, with limited recruitment and data collection. It consists of small sample size also due to the rarity of SSc, with monocentric study design and limited measurement parameters. This prevents generalization of the findings and associations might be masked or prone to bias. Specifically, the clinical settings, dietary factors and other fluctuations might increase variability of T50 (57) and associated factors. The reason for the inconsistent observations on FGF23 in the literature (24, 25, 49, 50) are currently unclear, but in theory small sample sizes, degradation of FGF23 in blood samples (58), diurnal variations and dietary factors could lead to larger variations. We had to conduct measurements of iFGF23 in serum, which is known to yield lower values (59). In addition, we could not determine other known or unknown factors that might directly or indirectly affect serum calcification propensity, such as vitamin D or pH status (27). Also, a possible influence on the mineral buffering system by drugs commonly used for treatment of SSc is currently unclear. Furthermore, our cross-sectional study is observational and does not allow to conclude on causality, and it is not possible to conclude whether the dysregulated mineral buffering system contributes to or results from SSc.

## Conclusion

In summary, this study provides the first indication for a deranged mineral buffering system with disturbed phosphate homeostasis in SSc. Increased serum calcification propensity (lower T50) is correlated with reduced pulmonary diffusion capacity in SSc patients. Further studies are required to confirm these observations in larger cohorts and to delineate a functional relevance in the course of the disease, since countering the deranged mineral buffering in SSc could be a hypothetical therapeutic strategy.

## Data Availability

The original contributions presented in the study are included in the article/**Supplementary Material**. Further inquiries can be directed to the corresponding author.
