# Supplementary material for: Accelerated calciprotein crystallization time (T50) is correlated with impaired lung diffusion capacity in systemic sclerosis
Source: Front Immunol. 2024 Sep 27;15:1425885. doi: 10.3389/fimmu.2024.1425885 (PMC11466802; doi:10.3389/fimmu.2024.1425885)
Supplement: Supplementary file 1 [file Table1.pdf]

Accelerated calciprotein crystallization time (T50) is correlated with impaired lung diffusion capacity in systemic sclerosis

Supplementary material

Supplemental tables

Suppl. Table S1: Patient characteristics

N numbers of SSc patients with documented characteristics and relevant medications.

	patients/total
PAH	14/78
Lung fibrosis	27/78
Dysphagy	39/78
Reflux	38/77
Raynaud	77/78
Digital ulcers	15/78
Sicca	33/74
Prednisolone	9/78
Mycophenolate mofetil	10/78
Tacrolimus	1/78
Ebetrexat	21/78
Ambrisentan	1/78
Macitentan	14/78
Sildenafil	7/78
Tadalafil	1/78
Bosentan	15/78
Treprostinil	3/78
Proton pump inhibitors	57/78
Calcium and/or Vitamin D	57/78

(PAH - pulmonary arterial hypertension)

Suppl. Table S2: Characteristics of control group

N numbers of control patients included in the study by reason for visits at the outpatient dermatology clinic.

	N
allergy	17
basalioma	14
alopecia	1
eczema	1
drug-induced intolerance	1
lipoma	1
psoriasis vulgaris	1
raynaud	1
scar	1
St.p. Anaphylaxis	1
St.p. Exanthema	1
St.p. Urticaria	1
St.p. Urticaria, St.p. Prostata Carcinoma	1
unclear gi symptoms	1
chronic urticaria	1

Suppl. Table S3: Associations of mRSS at baseline

Factors at baseline associated with modified Rodnan skin score (mRSS, 0-51, n=67, change in F p=0.044) in patients with SSc in a linear regression model, shown with regression coefficients (beta) and levels of significance (p value; bolded if p < 0.05).

mRSS [0-51]	beta	p
**Calcium [mmol]**	**-0.350**	**0.006**
Phosphate [mmol]	0.189	0.131
eGFR [ml/min/1.73m^2]	-0.120	0.402
Disease duration [yrs]	0.026	0.833
Body weight [kg]	-0.185	0.254
Body size [cm]	-0.180	0.311
Sex [m0/f1]	-0.256	0.111
Age [yrs]	-0.014	0.929
T50 [min]	0.029	0.862
CPP2 radius [nm]	0.137	0.406

Suppl. table S4: Associations of mRSS at follow-up

Factors at follow-up associated with modfied Rodnan skin score (mRSS, 0-51, n=46, change in F p=0.018) in patients with systemic sclerosis in a linear regression model, shown with regression coefficients (beta) and levels of significance (p value; bolded if p < 0.05).

Suppl. table S4

mRSS [0-51]	beta	p
Calcium [mmol]	-0.127	0.428
Phosphate [mmol]	0.169	0.302
eGFR [ml/min/1.73m^2]	-0.057	0.734
Disease duration [yrs]	0.098	0.476
Body weight [kg]	-0.272	0.130
Body size [cm]	-0.226	0.279
**Sex [m0/f1]**	**-0.617**	**0.001**
Age [yrs]	-0.026	0.884
T50 [min]	-0.217	0.181
CPP2 radius [nm]	0.100	0.483
